# 16S pan-bacterial PCR can accurately identify patients with ventilator-associated pneumonia

**DOI:** 10.1136/thoraxjnl-2016-209065

**Published:** 2016-12-14

**Authors:** Andrew Conway Morris, Naomi Gadsby, James P McKenna, Thomas P Hellyer, Paul Dark, Suveer Singh, Timothy S Walsh, Danny F McAuley, Kate Templeton, A John Simpson, Ronan McMullan

**Affiliations:** 1 Division of Anaesthesia, Department of Medicine, University of Cambridge, Cambridge, UK; 2 Centre for Inflammation Research, University of Edinburgh, Edinburgh, UK; 3 Department of Clinical Microbiology, NHS Lothian, Edinburgh, UK; 4 Department of Microbiology, Belfast Health & Social Care Trust, Belfast, UK; 5 Institute of Cellular Medicine, Newcastle University, Newcastle, UK; 6 Institute of Inflammation and Repair, University of Manchester, Manchester Academic Health Sciences Centre, Manchester, UK; 7 Intensive Care Unit, Salford Royal NHS Foundation Trust, Greater Manchester, UK; 8 Intensive Care Unit, Chelsea and Westminster Hospital, Imperial College London, London, UK; 9 Centre for Infection and Immunity, Queen's University Belfast, UK; 10 Intensive Care Unit, Royal Victoria Infirmary, Belfast, UK

**Keywords:** Pneumonia, Assisted Ventilation, Bacterial Infection

## Abstract

Ventilator-associated pneumonia (VAP) remains a challenge to intensive care units, with secure diagnosis relying on microbiological cultures that take up to 72 hours to provide a result. We sought to derive and validate a novel, real-time 16S rRNA gene PCR for rapid exclusion of VAP. Bronchoalveolar lavage (BAL) was obtained from two independent cohorts of patients with suspected VAP. Patients were recruited in a 2-centre derivation cohort and a 12-centre confirmation cohort. Confirmed VAP was defined as growth of >10^4^ colony forming units/ml on semiquantitative culture and compared with a 16S PCR assay. Samples were tested from 67 patients in the derivation cohort, 10 (15%) of whom had confirmed VAP. Using cycles to cross threshold (C_t_) values as the result of the 16S PCR test, the area under the receiver operating characteristic (ROC) curve (AUROC) was 0.94 (95% CI 0.86 to 1.0, p<0.0001). Samples from 92 patients were available from the confirmation cohort, 26 (28%) of whom had confirmed VAP. The AUROC for C_t_ in this cohort was 0.89 (95% CI 0.83 to 0.95, p<0.0001). This study has derived and assessed the diagnostic accuracy of a novel application for 16S PCR. This suggests that 16S PCR in BAL could be used as a rapid test in suspected VAP and may allow better stewardship of antibiotics.

**Trial registration number:**

VAPRAPID trial ref NCT01972425.

## Introduction

Ventilator-associated pneumonia (VAP) remains a significant problem in intensive care units (ICUs)^1^ and despite reductions in reported VAP rates antibiotic use remains high.^2^ The most common indication for antibiotic use remains suspected respiratory infections.^3^ VAP is associated with significant morbidity and mortality^1^ especially when antibiotics are delayed or are inadequate.^4^ However, due to the various conditions that can mimic VAP, commonly only 30% of those suspected of having VAP subsequently have this diagnosis confirmed.^4^ The delays in obtaining results from conventional microbiological cultures lead to empirical use of broad-spectrum antibiotics of which a significant proportion is later deemed unnecessary. The excessive use of antibiotics is associated with increased antimicrobial resistance^5^ and mortality.^6^

The ubiquitous presence of a 16S ribosomal RNA gene in bacteria offers the possibility of detecting a wide range of bacteria in a single PCR.^7^ Amplification of the 16S rRNA gene in a PCR assay results in amplification of all bacteria in a sample. Therefore, this offers potential as a screening test for suspected VAP. The aim of this study was to derive and validate a real-time 16S PCR assay for diagnosing confirmed VAP.

## Methods

Samples from two previously described^8^ ^9^ cohorts of adult patients with clinically suspected VAP recruited from UK ICUs formed the derivation^8^ and confirmation^9^ cohorts respectively. Briefly, patients were recruited if they met criteria for suspected VAP, namely new or worsening chest X-ray changes following at least 48 hours of ventilation, accompanied by two or more of: temperature >38°C; white cell count >11×10^9^/L; or mucopurulent sputum. In the derivation cohort patients were excluded if they had received new antibiotics within the 3 days prior to recruitment;^8^ no such exclusion was applied to the confirmation cohort.^9^ Patients underwent protocolised bronchoscopic bronchoalveolar lavage (BAL)^8^ ^9^ and an aliquot of BAL fluid was processed using a semiquantitative culture method. This culture was used as our reference diagnostic standard, with growth at >10^4^ colony forming units/mL (CFU/mL) of BAL fluid being defined as ‘VAP positive’ and growth <10^4^ CFU/mL as ‘VAP negative’, these cut-offs being in line with established standards.^1^ ^4^

Full details of sample processing are described in the online supplementary section. Briefly, the fraction of lavage not used for conventional culture was centrifuged to produce a cell-free supernatant, followed by nucleic acid extraction. The 16S PCR assays are described below; assay 1 and assay 2 were conducted in geographically separate laboratories.

10.1136/thoraxjnl-2016-209065.supp1 supplementary data

### Real-time 16S PCR assay 1

The primer and probe sequences targeting the16S rRNA gene have been described previously.^10^ The probe contained a carboxyfluorescein (FAM) label on the 5′ end with a Black Hole Quencher 1 (BHQ1) on the 3′ end. Primers and probe were synthesised by Eurogentec (Liège, Belgium). The final 16S PCR reaction mix contained 1.25U HotStarTaq polymerase and 1× reaction buffer (Qiagen, Manchester, UK), 4 µM MgCl_2_, 0.2 mM deoxynucleotide mix (dNTP), 0.25 µM primer 27-F, 0.75 µM primer 16S 1RR-B, 0.3 µM probe 514-S, nuclease-free water (Promega, Southampton, UK) and 10 µL nucleic acid extract to a final volume of 25 µL. Real-time PCR was carried out on the ABI 7500 instrument (Applied Biosystems, Life Technologies, Paisley, UK). This assay was used for samples from the derivation cohort, to establish proof in principle of the diagnostic utility of this approach, and was also used for samples from the confirmation cohort.

### Real-time 16S PCR assay 2

The primer and hybridisation probe sequences targeting the 16S rRNA gene have been described previously.^11^ The hybridisation probe contained a FAM label on the 5′ end with a BHQ1 on the 3′ end. Primers and hybridisation probe were synthesised by Sigma Genosys (Sigma-Aldrich, Ebersberg, Germany).

The final 16S PCR reaction mix contained 1X Platinum uracil DNA glycosylase Mastermix (Life Technologies, Paisley, UK), 0.2 µM bovine serum albumin (Sigma, Dorset, UK), a total of 4 mmol/L MgCl_2_, 0.4 µM forward and reverse primers, 0.1 µM hybridisation probe, nuclease-free water (Promega, Southampton, UK) and 2 µL of target template for a final reaction volume of 10 µL. Real-time qPCR was carried out on a Light Cycler 480 instrument (Roche, Indianapolis, Indiana, USA). This assay was used on samples from the confirmation cohort only.

For the purposes of analysis, the metric was cycles to cross threshold (C_t_) as a measure of 16 s rRNA gene load and hence bacterial burden. A higher bacterial load will result in a lower time to cross threshold, that is, a lower C_t_ value. Details of statistical analyses used can be found in the online supplementary methods section. Both studies had approvals from relevant research ethics committees; full details are in the online supplementary section.

## Results

In the derivation cohort, samples from 67 patients were available, of whom 10 (15%) had ‘microbiologically confirmed VAP’. In the ‘confirmation’ cohort samples from 92 patients were available for analysis; 26 (28%) met the culture criteria for ‘microbiologically confirmed VAP’. The demographic details and organisms cultured are shown in the online supplementary section (see online supplementary tables S1 and S2).

**Figure 1:**
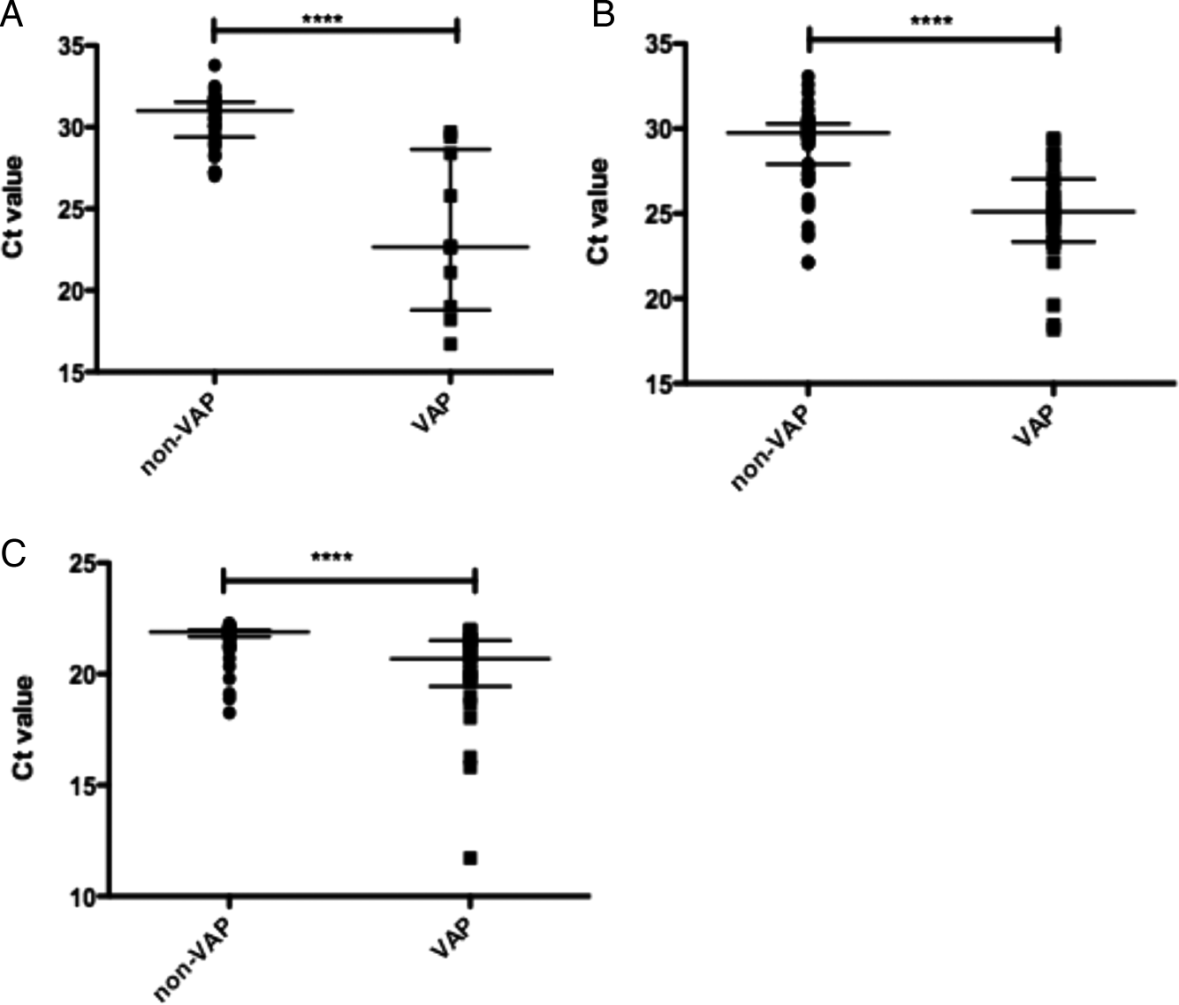
Real-time 16S PCR results as expressed by cycles to cross threshold (Ct) for samples from patients. (A) C_t_ values from assay 1 among derivation cohort patients with and without confirmed ventilator-associated pneumonia (VAP). N=67, 57 non-VAP and 10 VAP, error bars show median and IQR. **** p<0.0001 by Mann-Whitney U test. (B) C_t_ values from assay 1 among confirmation cohort patients with and without confirmed VAP. N=92, 66 non-VAP and 26 VAP, error bars show median and IQR. **** p<0.0001 by Mann-Whitney U test. (C) Ct values from assay 2 among confirmation cohort patients with and without confirmed VAP. N=92, 66 non-VAP and 26 VAP, error bars show median and IQR. **** p<0.0001 by Mann-Whitney U test.

16S PCR assay 1 demonstrated that patients with confirmed VAP had a higher bacterial burden, as signified by a lower C_t_ value, than those without VAP (figure 1A). When evaluated for diagnostic ability by ROC curve, assay 1 demonstrated excellent diagnostic ability (see table 1 and figure S1A) with an area under the ROC curve (AUROC) of 0.94 (95% CI 0.86 to 1.00), sensitivity of 100% and specificity 72% at the most optimal cut-off.

**Table 1:** Diagnostic performance of the two 16S assays

Curve	Assay 1 derivation	Assay 1 confirmation	Assay 2 confirmation
AUC ROC	0.94 (0.86 to 1.0) p<0.0001	0.89 (0.83 to 0.95) p<0.0001	0.84 (0.75 to 0.94) p<0.0001
Youden optimum cut-off (Ct)	29.85	29.43	21.59
Youden optimum sensitivity/specificity (95% CIs)	100 (69 to 100)/72 (58 to 83)	100 (87 to 99)/67 (54 to 78)	89 (70 to 98)/80 (69 to 89)
Maximum sensitivity optimum cut-off (Ct)	29.85	29.43	22.02
Maximum sensitivity/specificity (95% CIs)	100 (69 to 100)/72 (58 to 83)	100 (87 to 100)/67 (54 to 78)	100 (86 to 100)/15 (8 to 26)

(ROC curves displayed in online supplementary figure S1).

As avoiding false-negative results is important in rapid tests for VAP, we also report the specificity at maximum (100%) sensitivity.

AUC, area under the curve; C_t_, cycles to crossing threshold; VAP, ventilator-associated pneumonia.

In the confirmation cohort, patients with confirmed VAP had significantly lower 16S C_t_ values (figure 1B), and a similar diagnostic performance was demonstrated (table 1 and figure S1B), with sensitivity of 100% and specificity of 67% at the most optimal cut-off. The difference between the AUROC of the cohorts was not statistically significant (p=0.56).

Samples from the confirmation cohort were also tested using 16S assay 2. As seen in figure 1C, although the absolute C_t_ values differed between the two assays, the same relationship between VAP and non-VAP samples was observed. ROC analysis (table 1 and figure S1C) demonstrated good diagnostic ability (area under the curve 0.84 95% CI 0.75 to 0.94) with sensitivity 89% and specificity 80% at the optimal cut-off. Although the point estimates of AUROC were higher for assay 1, the difference did not achieve statistical significance (p=0.4). However if the assays are compared at maximal sensitivity (100%), the specificity of assay 1 is significantly higher (table 1). Using the Youden Index to define optimal C_t_ value cut-offs on the ROC curve, a ‘positive’ result for 16S would be a value below this cut-off (indicating high bacterial load) and a ‘negative’ result would be a value above this cut-off (indicating low bacterial load).

In the derivation cohort, 35 (52%) patients were receiving antibiotics on the day of recruitment. In the confirmation cohort, 69 (75%) were receiving antibiotics and 14 (15%) had undergone a change of antibiotics within the past 3 days. Receipt of antibiotics and recent change in antibiotics were not associated with changes in 16S C_t_ values (see online supplementary results and table S3).

Figure S2 shows the relationship between C_t_ values for the two 16S assays, demonstrating a non-linear association.

## Discussion

To our knowledge, this is the first report of the use of real-time 16S PCR for diagnosing VAP. Although 16S rRNA gene sequencing has been used to explore the microbiome of ventilated patients, data on its diagnostic potential have been absent. In deriving and confirming a test, with a high agreement in test performance between the two cohorts, we demonstrate clear potential for the clinical utility of this test. Turnaround time is 4–6 hours; therefore, this test could impact on antibiotic use, which may otherwise only be rationalised following the results of conventional cultures at 48–72 hours.

This study has a number of strengths. First, we were able to perform derivation and confirmation in two distinct cohorts, with confirmation in a cohort recruited from a diverse group of 12 ICUs. The results are therefore likely to be widely applicable; indeed, the microbiological spectrum found is similar to reports from other countries.^4^ Second, by using consistent diagnostic procedures within each cohort, we avoided some of the problems which occur with the diagnosis of VAP.^1^ ^4^ Our rate of microbiologically confirmed VAP in both cohorts (23%) is at the lower end of the reported range^4^ but not out of keeping with other reports and we believe this may, in part, reflect the use of highly standardised BAL protocols.

A disadvantage of this study is that samples were obtained bronchoscopically, requiring resource and exposing patients to a small but definite risk, and the applicability of this test to other sample types cannot be inferred. The assays we describe here are also limited to bacterial detection. The differences between the two assays tested, and the use of stored samples, highlight the need for external prospective validation before this measure could be implemented in routine clinical practice. Further refinements of assays may also improve diagnostic performance. The reference standard of growth of organisms on conventional culture, remains imperfect, and indeed may well be influenced by intercurrent antibiotics generally, and recent changes in antibiotics specifically.^12^ However this remains the established standard^4^ and is used routinely for clinical decision-making. As such, the 16S assay described here can predict the results of a clinically relevant test, but within 6 hours rather than the 48–72 hours taken for the conventional cultures.

In conclusion, we have derived and confirmed the diagnostic utility of a rapid laboratory test for VAP in a multicentre setting. We propose that this test has the potential to permit rapid decisions to direct antimicrobial therapy in patients with suspected VAP thus improving stewardship of antibiotics in the ICU.
