# Supplementary material for: 16S pan-bacterial PCR can accurately identify patients with ventilator-associated pneumonia
Source: Thorax. 2016 Dec 14;72(11):1046–8. doi: 10.1136/thoraxjnl-2016-209065 (PMC5738539; doi:10.1136/thoraxjnl-2016-209065)
Supplement: supplementary data [file thoraxjnl-2016-209065supp001.pdf]

Supplemental methods

Semi-quantitative culture of BAL fluid

After thorough mixing, 10μl of whole BAL fluid was inoculated and evenly spread onto solid culture media and incubated for 40-48 hours. The resulting number of colonies of bacterial pathogens was counted, with fewer than 10 taken to indicate <10^3 colony-forming units (CFU) per mL, 10-100 colonies indicating 10^3-10^4 CFU/mL, and greater than 100 colonies indicating >10^4 CFU/mL. Confirmed VAP was defined as growth of bacteria at >10^4 CFU/ml [1,4].

Sample processing

BAL fluid was prepared as described previously [8,9] with the cell-free supernatant being stored at -80°C. BAL fluid from the derivation cohort underwent nucleic acid extraction using the DNeasy Blood and Tissue kit (Qiagen, Manchester, UK) with a pre-treatment protocol for Gram positive bacteria according to the manufacturer's instructions. Nucleic acid extraction on samples from the validation cohort was completed using MagNA pure 96 DNA and viral NA small volume kit (Roche, Indianapolis, IN, USA), by different staff, in a separate laboratory. Negative control samples comprising sterile saline underwent identical extraction to give a measure of 'background' 16S rRNA gene DNA in assay reagents.

Statistical analysis

Non-normal data are presented as median and inter-quartile range, and analysed by Mann-Whitney U- test. The diagnostic performance of the 16S assays, expressed as cycle number to cross threshold (C_t) was analysed by plotting Receiver Operator Characteristic (ROC) curves, with optimum cut-off defined by the Youden index (the cut off which produces the largest sum of sensitivity and specificity)[S2]. Differences between areas under ROC curves were analysed by the methods of Hanley and McNeil[S3,S4]. P<0.05 was taken to indicate statistical significance. Analysis was conducted using Prism (v5f for Mac, Graphpad, Carlsbad, CA, USA).

Ethical permissions

All procedures performed in studies involving human participants were in accordance with the ethical standards of the institutional and/or national research committee and with the 1964 Helsinki declaration and its later amendments or comparable ethical standards. The samples from patient derivation and validation cohorts were collected in studies approved by Lothian Research Ethics Committee (REC) (LREC/2002/8/19) and NRES North East REC (11/NE/0242) and Scotland A REC (11/SS/0089), respectively, with informed consent/assent from the next of kin.

Supplemental results

Characteristic	Derivation cohort	Validation
N=	67	92
Mean Age (range) years	59 (26-87)	60 (18-87)
% male	65%	71%
Median (IQR) APACHE II Score on admission	21 (16-26)	20 (15-23)
% Surgical admission	50%	41%
Median (IQR) ICU length of stay	23 (15-30)	19 (12-35)
% ICU mortality	28%	28%
% Hospital mortality	33%	37%
% Receiving antibiotic at time of lavage	52%	75%
% undergoing change in antibiotic therapy within 3 days of enrolment	0%	15%

Table S1. Demographic and clinical features of the derivation and validation cohorts.

Type of organism	Derivation	Validation
Enterobacteriaceae	**4**	**9**
Haemophilus spp. & Moraxella spp.	**1**	**2**
Pseudomonas aeruginosa	**0**	**5**
Staphylococcus aureus	**4**	**8**
**Other bacteria** (*Streptococcus* spp., *Acinetobacter baumannii*, coagulase-negative *Staphylococcus* spp.)	**1**	**4**

Table S2: Bacteria grown at >10^4 CFU/ml in the derivation and validation cohorts. Two patients in the validation cohort grew more than one organism above the threshold.

Influence of antibiotics on results of the 16s assay

In the derivation cohort, 35 (52%) of patients were receiving antibiotics on the day of recruitment, and none had experienced a change in antibiotics in the preceding three days. In the validation cohort, 69 (75%) of patients were receiving antibiotics on the day of enrolment. As can be seen in table S3 below, there were no significant differences in C_t values between patients receiving antibiotics, and those not receiving antibiotics, in either assay. Recent change in antibiotics is more likely to create 'false negatives' on conventional cultures[11], and (15%) of validation cohort patients had a change of antibiotics in the preceding three days. Again we found no difference in C_t values between these groups, on either assay.

Cohort	Not receiving antibiotics on enrolment	Receiving antibiotics on enrolment	P value	Change of antibiotics within 3 days	No change of antibiotics within 3 days	P value
Derivation	30.5(22.7-31.4)	31.0(28.3-31.5)	0.96	NA	NA	
Validation – assay 1	27.1 (26.2-30.1)	29.5 (26.2-30.1)	0.13	29.5 (27.4-30.0)	29.1 (25.6-30.1)	0.63
Pooled derivation and validation-assay 1	29.5 (26.0-31.2)	29.7 (27.3-30.4)	0.96	NA	NA	
Validation – assay 2	21.9(21.3-22.0)	21.5 (20.4-21.9)	0.23	21.8 (20.9-21.9)	21.9(21.4-22.0)	0.25

Table S3. C_t values for patients by antibiotic status at time of study recruitment. Values shown as median (interquartile range), p value from Mann-Whitney U test comparing the preceding two columns.

Supplemental References

Additional references used solely in the supplemental section are indicated below

S1 Yang S, Lin S, Kelen GD, et al. Quantitative multiprobe PCR assay for simultaneous detection and identification to species level of bacterial pathogens. *J Clin Microbiol*. 2002;40:3449–3454.

S2 Youden WJ. Index for rating diagnostic tests. *Cancer*. 1950;3:32–35.

S3 Hanley JA, McNeil BJ. The meaning and use of the area under a receiver operating characteristic (ROC) curve. *Radiology*. 1982;143:29-36.

S4 Hanley JA, McNeil BJ. A method of comparing the areas under receiver operating characteristic curves derived from the same cases. *Radiology*. 1983;148:839-843.

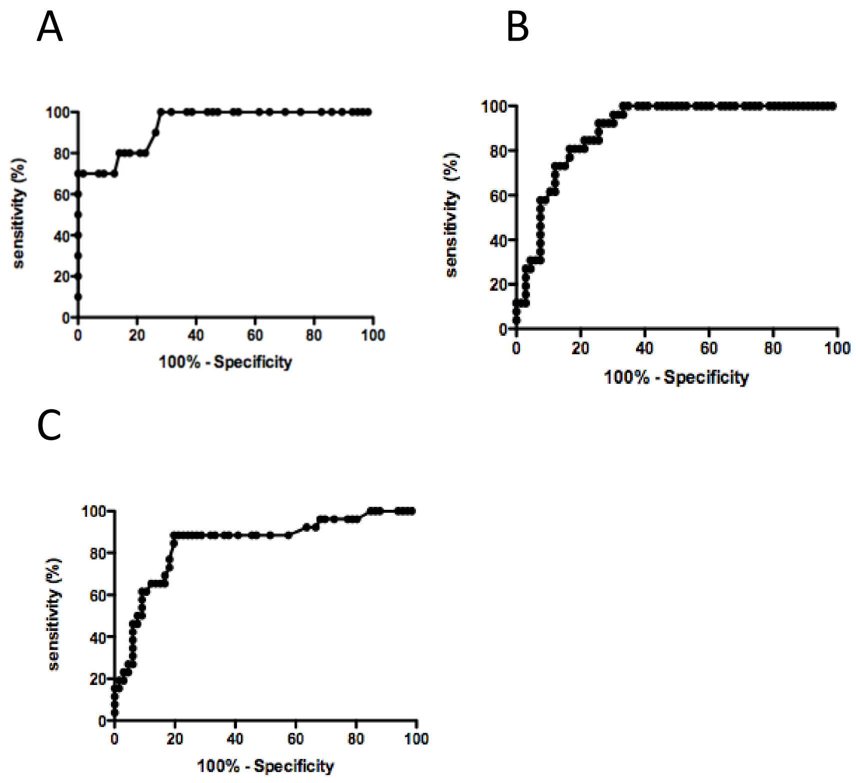

Figure S1: ROC curves for real-time 16S assays. Details of test performance are shown in table 1 of the main manuscript.

Panel A. ROC from assay 1 amongst derivation cohort patients with and without confirmed VAP.

Panel B. ROC from assay 1 amongst validation cohort patients with and without confirmed VAP.

Panel C. ROC from assay 2 amongst validation cohort patients with and without confirmed VAP.

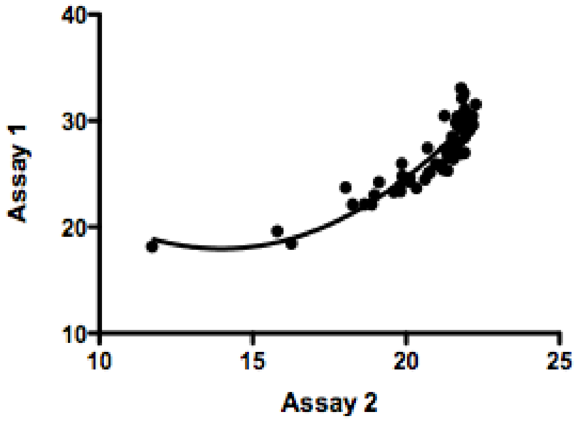

Figure S2: Relationship between results from real-time 16S PCR assays 1 and 2 on the validation cohort samples. The regression line describes a non-linear function ($y=28.96+3.38xX+0.06xX^2$), $r^2=0.91$, results from $n=92$ samples.
